# Supplementary material for: A DNase Type VI Secretion System Effector Requires Its MIX Domain for Secretion
Source: Microbiol Spectr. 2022 Sep 13;10(5):e02465-22. doi: 10.1128/spectrum.02465-22 (PMC9602870; doi:10.1128/spectrum.02465-22)
Supplement: Supplemental file 1 — Download spectrum.02465-22-s0001.pdf, PDF file, 0.8 MB [file spectrum.02465-22-s0001.pdf]

Supplementary Information

A DNase Type VI Secretion System Effector Requires Its MIX Domain for Secretion

Supplementary Figures

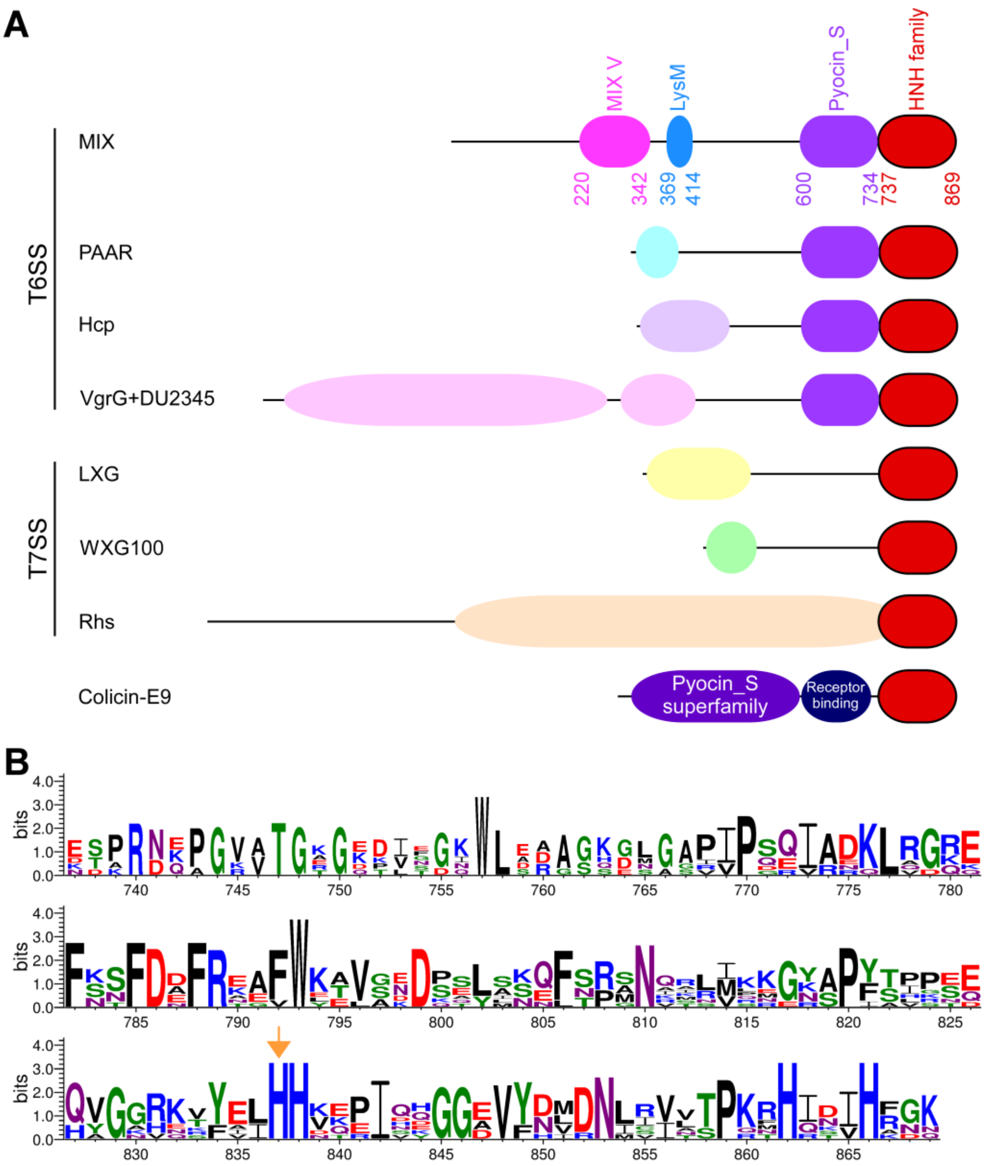

Supplementary Figure S1. Homology and conservation of VPA1263's C-terminal toxin domain. (A) Schematic representation of selected homologs of the C-terminal toxin domain in VPA1263. N-terminal domains and predicted secretion systems are denoted on the left. Secretion systems were determined by system-specific domains or neighboring genes. MIX, VPA1263 from *Vibrio parahaemolyticus* RIMD 2210633; PAAR, PQQ40857.1 from *Photorhabdus luminescens* H5; Hcp, SMR99238.1 from *Vibrio mangrovi* CECT 7927; VgrG, EAV0570772.1 from *Salmonella enterica* PNUSAS065855; LXG, EOP65835.1 from *Bacillus cereus* VD118; WXG100, EJP82933.1 from *Bacillus cereus* VD022; Rhs, REH35728.1 from *Kutzneria buriramensis* DSM 45791; Colicin-E9, CAA31104.1 from *Escherichia coli* k-12. **(B)** A conserved motif of the VPA1263 C-terminal HNH family domain, as illustrated using WebLogo 3, based on multiple sequence alignment of the proteins described in **A**. The numbers below correspond to the positions in VPA1263. An orange arrow denotes the active site Histidine at position 837 of VPA1263.

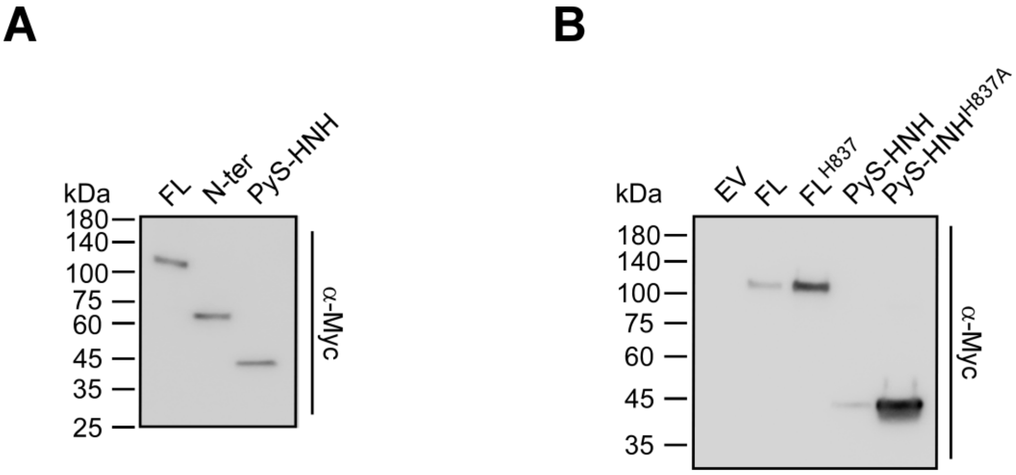

Supplementary Figure S2. Expression of VPA1263 variants in *E. coli*. (A-B) Expression of the indicated C-terminal Myc-His-tagged VPA1263 variants from an arabinose-inducible plasmid in *E. coli* MG1655. Proteins were detected by immunoblotting using specific α-Myc antibodies. FL, full-length VPA1263; N-ter, VPA1263 amino acids 1-546; PyS-HNH, VPA1263 amino acids 547-869; EV, empty plasmid; FL^{HA}, $VPA1263^{H837A}$; PyS-HNH^{H837A}, VPA1263 amino acids 547-869 with a H837A mutation.

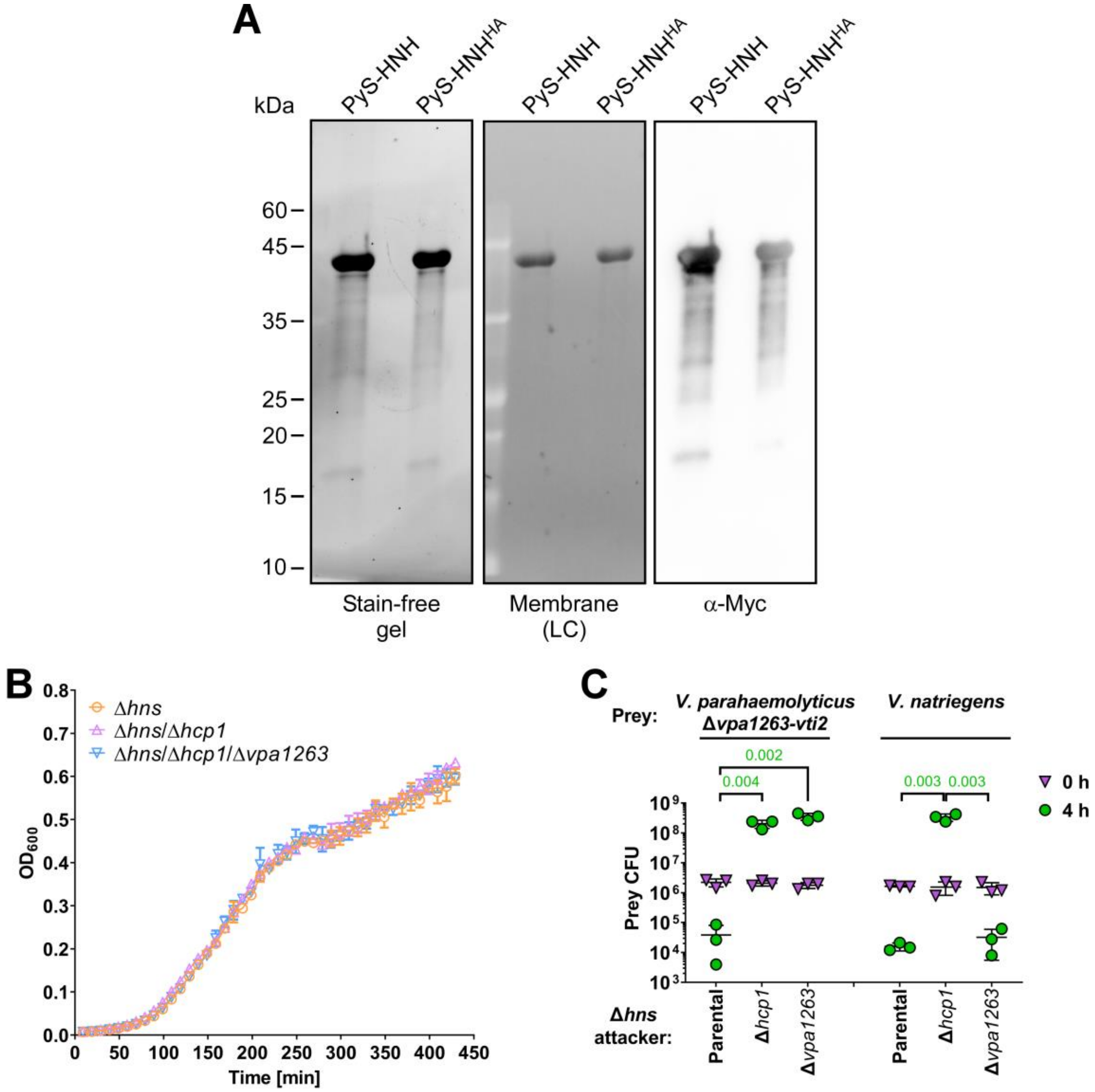

Supplementary Figure S3. VPA1263 mediates T6SS-dependent killing. (A) Purification of $VPA1263^{547\text{-}869}$ (PyS-HNH) and $VPA1263^{547\text{-}869/H837}$ ($PyS\text{-}HNH^{HA}$) containing a C-terminal Myc-His tag from *E. coli*. Final elution fractions were resolved on TGX stain-free gels (Bio-Rad); total proteins were visualized using the gel's trihalo compounds' fluorescence after UV activation on the gel and after transfer to a nitrocellulose membrane (LC, loading control). VPA1263 variants were detected by immunoblotting with α-Myc antibodies. **(B)** Growth of the indicated *V. parahaemolyticus* RIMD 2210633 derivative strains in MLB at 30°C is shown as optical density measurements at 600 nm (OD_{600}). Data are shown as the mean ± SD, $n = 3$ technical repeats. The experiment was performed three times with similar results; results of a representative experiment are shown. **(C)** Viability counts of the indicated prey strains before (0 h) and after (4 h) co-incubation with the indicated *V. parahaemolyticus* RIMD 2210633 Δ*hns* derivative attacker strain on MLB agar plates at 30°C. Prey strains contain an empty plasmid that provides a selection marker. Data are shown as the mean ± SD, $n = 3$ technical replicates. Statistical significance between samples at the 4 h time point by an unpaired two-tailed Student's *t*-test is denoted above. The experiment was performed three times with similar results; results of a representative experiment are shown.

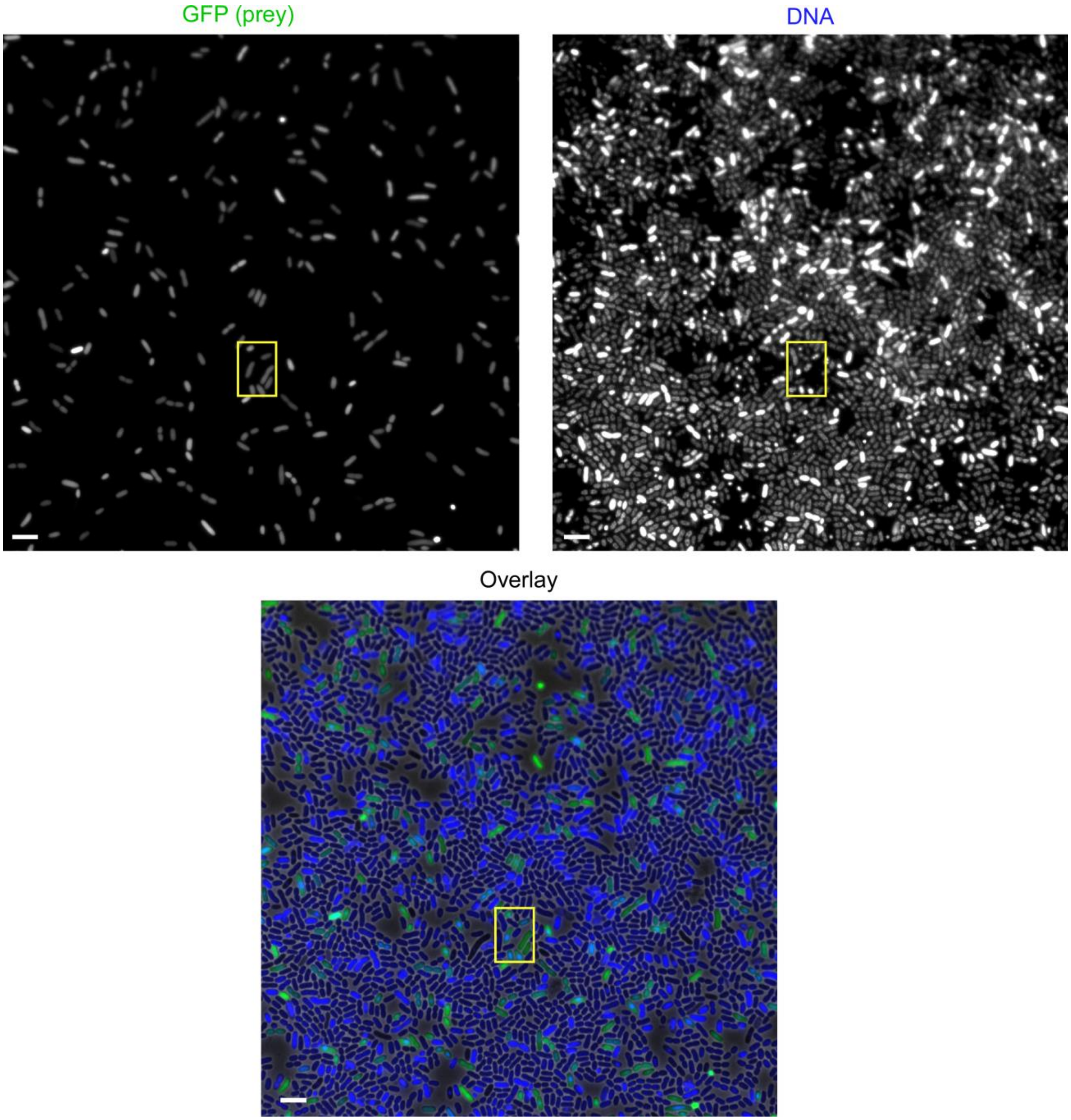

Supplementary Figure S4. VPA1263-mediated toxicity results in aberrant prey DNA morphology during bacterial competition. The complete field of view of fluorescence microscope images shown in Figure 2D. The section shown in Figure 2D is denoted with a yellow rectangle. Bar = 4 µm.

Supplementary Tables

Supplementary Table S1. A list of bacterial strains used in this study.

Strain name	Genotype	Comments	Source
Vibrio parahaemolyticus RIMD 2210633	Wild type	Used for generating deletion strains and as a template for PCR amplification	Obtained from Kim Orth; (1)
Vibrio parahaemolyticus RIMD 2210633 Δ*hns*	Δ*hns*	A RIMD 2210633 derivative containing an in-frame deletion of *vp1133*; it is used as an attacker in bacterial competition assays, and in secretion assays	(2)
Vibrio parahaemolyticus RIMD 2210633 Δ*hns*/Δ*hcp1*	Δ*hns*/Δ*hcp1*	A RIMD 2210633 derivative containing an in-frame deletion of *vp1133* and of *vp1393*; it is used as an attacker in bacterial competition assays and in secretion assays	(2)
Vibrio parahaemolyticus RIMD 2210633 Δ*hns*/Δ*vpa1263*	Δ*hns*/Δ*vpa1263*	A RIMD 2210633 derivative containing an in-frame deletion of *vp1133* and of *vpa1263*; it is used as an attacker in bacterial competition assays	This study

Strain name	Genotype	Comments	Source
Vibrio parahaemolyticus RIMD 2210633 Δ*vpa1263-vti2*	Δ*vpa1263-vti2*	A RIMD 2210633 derivative containing an in-frame deletion of *vpa1263-vti2*; it is used as a prey in bacterial competition assays	This study
Vibrio parahaemolyticus POR1 $vp1415^{AAA}$	Δ*tdhAS*/$vp1415^{AAA}$	A RIMD 2210633 derivative. It is used as a template for PCR amplifications. Codons for histidines 563-4 in VP1415 were replaced by codons for alanines	(3)
Vibrio natriegens ATCC 14048	Wild type	Used as a prey in competition assays	ATCC collection
Escherichia coli DH5α (λ pir)	K-12 derivative laboratory strain containing λ pir	Used for plasmid construction and maintenance	Obtained from Eric V. Stabb
Escherichia coli MG1655	Wild type	Used for protein expression, toxicity assays, and in vivo DNase assays	Lab stocks
Escherichia coli BL21 (DE3)	Laboratory strain	Used for protein expression	Lab stocks

Supplementary Table S2. A list of plasmids used in this study.

Plasmid name	Description	Comments	Source
pDM4	a Cm^R and oriVR6K-containing suicide vector	Used to generate deletions in *Vibrio parahaemolyticus*	(4)
pDM4:*vpa1263*	pDM4 containing 1 kb upstream and 1 kb downstream of *vpa1263* in its MCS	Used to delete *vpa1263* in *V. parahaemolyticus* RIMD 2210633	(5)
pDM4:*vpa1263-vti2*	pDM4 containing 1 kb upstream of *vpa1263* and 1 kb downstream of *vti2* in its MCS	Used to delete *vpa1263-vti2* in *V. parahaemolyticus* RIMD 2210633	(5)
pDM4:*hns*	pDM4 containing 1 kb upstream and 1 kb downstream of *vp1133* (*hns*) in its MCS	Used to delete *vp1133* in *V. parahaemolyticus* RIMD 2210633	(6)
pBAD^K/Myc-His	pBR322 ori-containing plasmid harboring a Kan^R cassette, *araC*, and an MCS following a P*bad* promoter	Used for arabinose-inducible expression	(7)
pPoNe^{BC}	pBAD^K/Myc-His plasmid containing the CDS of BC3021 (PoNe^{BC}) from *Bacillus cereus* ATCC 14579, in frame with a C-terminal Myc-His tag	Used for arabinose-inducible expression of PoNe^{BC} in *E. coli*	(8)
pVPA1263	pBAD^K/Myc-His plasmid containing the CDS of *vpa1263,* in frame with a C-	Used for arabinose-inducible expression of VPA1263 (FL)	This study

Plasmid name	Description	Comments	Source
	terminal Myc-His tag	during toxicity assays in *E. coli*	
pVPA1263$^{1-546}$	pBAD^{K}/Myc-His plasmid containing the CDS of the N-terminal part of *vpa1263* (amino acids 1-546), in frame with a C-terminal Myc-His tag	Used for arabinose-inducible expression of VPA1263$^{1-546}$ (N-ter) during toxicity assays in *E. coli* and during secretion assays in *Vibrio*	This study
pVPA1263$^{547-869}$	pBAD^{K}/Myc-His plasmid containing the CDS of the C-terminal part of *vpa1263* (amino acids 547-869), in frame with a C-terminal Myc-His tag	Used for arabinose-inducible expression of VPA1263$^{547-869}$ (PyS-HNH) during toxicity assays and in vivo DNase assays in *E. coli*, and for protein purification	This study
pVPA1263$^{737-869}$	pBAD^{K}/Myc-His plasmid containing the CDS of the C-terminal HNH nuclease domain of VPA1263 (amino acids 737-869), in frame with a C-terminal Myc-His tag	Used for the arabinose-inducible expression of VPA1263$^{737-869}$ (HNH) during toxicity assays in *E. coli*	This study
pVPA1263^{H837A}	pBAD^{K}/Myc-His plasmid containing the CDS of *vpa1263* carrying a mutation in the active site H837A, in frame with a C-	Used for the arabinose-inducible expression of VPA1263^{H837A} (FL^{HA}) during toxicity assays in *E. coli* and during	This study

Plasmid name	Description	Comments	Source
	terminal Myc-His tag	secretion assays in *Vibrio*	
pVPA1263$^{\Delta MIX/H837A}$	pBAD^{K}/Myc-His plasmid containing the CDS of *vpa1263* lacking most of the MIX domain (amino acids 226-328) and carrying a mutation in the active site, H837A, in frame with a C-terminal Myc-His tag	Used for the arabinose-inducible expression of VPA1263$^{\Delta MIX/H837A}$ (ΔMIX^{HA})during secretion assays in *Vibrio*	This study
pVPA1263$^{547\text{-}869/H837A}$	pBAD^{K}/Myc-His plasmid containing the CDS of the C-terminal part of *vpa1263* (amino acids 547-869) and carrying a mutation in the active site H837A, in frame with a C-terminal Myc-His tag	Used for the arabinose-inducible expression of VPA1263$^{547\text{-}869/H837A}$ (PyS-HNH^{HA}) during toxicity assays and in vivo DNase assays in *E. coli*, for protein purifications, and during secretion assays in *Vibrio*	This study
pVPA1263$^{G247A/H837A}$	pBAD^{K}/Myc-His plasmid containing the CDS of *vpa1263* carrying a mutation in the MIX domain, G246A, followed by a mutation in the active site, H837A, in frame	Used for the arabinose-inducible expression of VPA1263$^{G246A/H837A}$ (FL$^{GA/HA}$) during secretion assays in *Vibrio*	This study

Plasmid name	Description	Comments	Source
	with a C-terminal Myc-His tag		
pVPA1263$^{Y250A/H837A}$	pBAD^{K}/Myc-His plasmid containing the CDS of *vpa1263* carrying a mutation in the MIX domain, Y250A, followed by a mutation in the active site, H837A, in frame with a C-terminal Myc-His tag	Used for the arabinose-inducible expression of VPA1263 $^{Y250A/H837A}$ (FL$^{YA/HA}$) during secretion assays in *Vibrio*	This study
pBAD33.1^{F}	pBAD33.1 with a FLAG tag inserted at the 3' end of the MCS	Used for arabinose-inducible expression	(9)
pVti2	pBAD33.1^{F} plasmid containing the CDS of *vti2,* in frame with a C-terminal FLAG tag	Used for the arabinose-inducible expression of Vti2 during toxicity assays in *E. coli*, and during protein purification	This study
pGFP	a Spec^{R}-containing, high copy number plasmid for the constitutive expression of GFP in vibrios	Used for the constitutive expression of GFP in *V. parahaemolyticus* prey visualized under a fluorescence microscope	(10)

Plasmid name	Description	Comments	Source
pCLTR	*E. coli*-yeast-*Vibrio* shuttle vector; mobilizable; it contains $Spec^R$ and Cm^R	Used to selectively grow *V. natriegens* prey on media containing chloramphenicol during bacterial competition	(3)
pVPA1263-Vti2	$pBAD^K$/Myc-His plasmid containing the CDS of *vpa1263* and *vti2*; *vti2* is in frame with a C-terminal Myc-His tag	Used for the arabinose-inducible expression of VPA1263 and Vti2 during bacterial competition assays	(3)
$pVPA1263^{G247A}$-Vti2	pVPA1263-Vti2 in which the codon for glycine 247 in *vpa1263* was replaced with a codon for alanine	Used for the arabinose-inducible expression of $VPA1263^{G247A}$ and Vti2 during bacterial competition assays	This study
$pVPA1263^{H837A}$-Vti2	pVPA1263-Vti2 in which the codon for histidine 837 in *vpa1263* was replaced with a codon for alanine	Used for the arabinose-inducible expression of $VPA1263^{H837A}$ and Vti2 during bacterial competition assays	This study

Supplementary References

1. Makino K, Oshima K, Kurokawa K, Yokoyama K, Uda T, Tagomori K, Iijima Y, Najima M, Nakano M, Yamashita A, Kubota Y, Kimura S, Yasunaga T, Honda T, Shinagawa H, Hattori M, Iida T. 2003. Genome sequence of Vibrio parahaemolyticus: a pathogenic mechanism distinct from that of V cholerae. Lancet 361:743–749.

2. Dar Y, Jana B, Bosis E, Salomon D. 2021. A binary effector module secreted by a type VI secretion system. EMBO Rep e53981.

3. Jana B, Keppel K, Salomon D. 2021. Engineering a customizable antibacterial T6SS-based platform in Vibrio natriegens. EMBO Rep 22:e53681.

4. O'Toole R, Milton DL, Wolf-Watz H. 1996. Chemotactic motility is required for invasion of the host by the fish pathogen Vibrio anguillarum. Mol Microbiol 19:625–637.

5. Salomon D, Kinch LN, Trudgian DC, Guo X, Klimko JA, Grishin N V., Mirzaei H, Orth K. 2014. Marker for type VI secretion system effectors. Proc Natl Acad Sci 111:9271–9276.

6. Salomon D, Klimko JA, Orth K. 2014. H-NS regulates the Vibrio parahaemolyticus type VI secretion system 1. Microbiol (United Kingdom) 160:1867–1873.

7. Salomon D, Gonzalez H, Updegraff BL, Orth K. 2013. Vibrio parahaemolyticus Type VI secretion system 1 Is activated in marine conditions to target bacteria, and is differentially regulated from system 2. PLoS One 8:e61086.

8. Jana B, Fridman CM, Bosis E, Salomon D. 2019. A modular effector with a DNase domain and a marker for T6SS substrates. Nat Commun 10:3595.

9. Fridman CM, Keppel K, Gerlic M, Bosis E, Salomon D. 2020. A comparative genomics methodology reveals a widespread family of membrane-disrupting T6SS effectors. Nat Commun 11:1085.

10. Ritchie JM, Rui H, Zhou X, Iida T, Kodoma T, Ito S, Davis BM, Bronson RT, Waldor MK. 2012. Inflammation and Disintegration of Intestinal Villi in an Experimental Model for Vibrio parahaemolyticus-Induced Diarrhea. PLoS Pathog 8:e1002593.
